# Supplementary material for: Identification of high-risk contact areas between feral pigs and outdoor-raised pig operations in California: Implications for disease transmission in the wildlife-livestock interface
Source: PLoS One. 2022 Jun 28;17(6):e0270500. doi: 10.1371/journal.pone.0270500 (PMC9239460; doi:10.1371/journal.pone.0270500)
Supplement: S1 Appendix — (PDF) [file pone.0270500.s001.pdf]

Agriculture Natural Resources (ANR), Cooperative Extension (UCCE) and UC Davis School of Veterinary Medicine are conducting a research study consisting of a voluntary survey designed specifically for owners of outdoor-raised domestic pigs in California

Background: In the last few decades, there has been an expansion of outdoor-raised (i.e., pasture-based) pig operations in the United States. This trend is driven by consumer demand for sustainably produced, humanely-raised, high-quality local meat products. However, the total numbers of pasture-raised pig operations are unknown.

One of the challenges of raising pigs outdoors is the possibility of domestic swine interfacing with wildlife, like feral pigs (aka feral swine, feral hogs, wild pigs, wild boars, etc.). Feral pigs can have a negative impact on the health of domestic swine raised outdoors.

This survey is appropriate for anyone that raises at least one domestic pig outside* including: pork producers, farmers, ranchers, backyard operations, 4-H/FFA members, pet pig owners, heritage pig breeders, pig rescue groups, etc.
(*also referred to as pasture-raised pigs, outdoor-raised pigs, i.e. pig(s) spend at least 50% or more of their time outside, not confined inside a barn or building)

Your participation will provide critical information for a research project to study the distribution of outdoor-raised domestic swine (i.e., pasture-raised) in California.

This survey should take **10-15 minutes** to complete and you'll have a chance to win a gift card. At the end of the survey, you can provide your email address and be given a chance to win one of three $100 Visa gift cards. You may also enter to win a gift card by providing only your email at the end of the online survey and clicking submit.

Your answers will help University of California Cooperative Extension (UCCE) develop outreach and educational materials for all producers that raise pigs outdoors. All provided information is kept confidential and only reported as a group (i.e. county level statistics), not on an individual/farm level. We will not connect your name nor location with your responses and we will not share this information with anyone not involved in this study. ***The information supplied by you on this survey will be treated strictly as confidential and used for statistical and spatial analysis purposes only.***

This survey is voluntary and is being conducted for research and outreach purposes only. You can choose not to participate and can quit the survey at any time. There will be no penalty or loss of services or benefits if you decide not to take part in the survey. This research has been reviewed and approved by an Institutional Review Board #1180798-1

Dr. Alda Pires and her lab staff appreciate your cooperation. If you have any questions or comments, please contact Alda Pires at apires@ucdavis.edu or Laura Paterson at lpatters@ucdavis.edu

University *of* California
Agriculture and Natural Resources

UC DAVIS
VETERINARY MEDICINE

A. General Livestock:

1) **How would you describe the reason(s) why you raise pig(s):** (Mark all that apply)
   a. **4-H or FFA**
   b. **Backyard producer:** raise pigs for personal or family consumption
   c. **Raise meat for commercial sale:** only raise pigs
   d. **Raise meat for commercial sale:** raise multiple types of livestock and/or poultry
   e. **Mixed crop-livestock farmer:** grows produce and raises pigs only
   f. **Mixed crop-livestock farmer:** grows produce and raises multiple types of livestock and/or poultry
   g. **Cheese maker/Creamery/Dairy** (raises pigs to dispose of whey, etc.)
   h. **Raise commercial nut or fruit trees** and raise pigs
   i. **Commercial vineyard** and raise pigs
   j. **Breeder** for:
      i. 4-H or FFA
      ii. Heritage breeds
      iii. Pet pigs
      iv. Commercial meat sales
      v. Other
   k. As a Pet
   l. Hobby/Rescue
   m. Other ____________________

2) **Which of the following <u>best</u> describes your role in raising outdoor-based domestic pigs:** (Choose the best answer)
   a. Owner
   b. Manager/Herdsman
   c. Employee/Staff
   d. Other______

3) How many pigs do you raise on average per year?

a. Adults (sows, gilts, boars) #________________
b. Juveniles (less than 1 year old) #_____________
c. Piglets #_______________
d. Total (average) herd size per year #___________

4) Do you raise your outdoor pig(s): (Mark all that apply)

a. On permanent pasture
b. On other non-pasture areas, like crop fields or orchards
c. In wild areas (forests, wetlands, etc.)
d. With access to natural surface water (ponds, creeks, reservoirs, etc.)
e. In pens outdoor that are mostly soil with no vegetative cover
f. In rotation (for instance rotate in crop fields or rotate pastures)
g. In an indoor pen that has access to outdoor areas/paddock

5) How many hours, on a typical day, do your pig(s) spend outside (not housed in a barn or enclosed structure)?

a. # Hours: ____________ (whole numbers only 1-24)

6) Do the areas where you raise your pig(s) border any of these environments? (Mark all that apply)

a. Wild areas
    i. Forests
    ii. Riparian area
    iii. Nature preserve or park
    iv. Other: ________________
    v. none
b. Surface water

i. Pond(s)/
ii. Reservoir(s)
iii. Lake(s)
iv. Creek(s)
v. River(s)
vi. Canal(s)
vii. Other: ________________
viii. None

7) Do you raise other types of livestock or poultry?

(place check mark ☑ under average number raised per year)

	1-45 animals	46-100 animals	101-150 animals	151-200 animals	201-300 animals	301-400 animals	Over 400 animals
**Layer Chickens**							
**Broiler Chickens**							
**Turkeys/Ducks/Geese**							
**Beef Cattle**							
**Dairy Cattle**							
**Goats**							
**Sheep**							
**Horses/Mules/Donkeys**							

B. Feral pigs: (aka feral swine, feral hogs, wild pigs, wild boars, etc.)

8) Have you seen evidence of feral pigs (e.g. scat, tracks, actual pigs, rooting in gardens, crops, wild areas, etc.): (Mark all that apply)

a) In outdoor-raised pig areas
b) In pens/barns/pig housing
c) On your farm/ranch/land
d) In your town/city/village
e) In your county, more than 2 miles from your farm
f) None

9) **Have you (or your employees) seen feral pigs within a mile or less of your land or outdoor-raised pigs?** (Choose the best answer)

a) Within the last week
b) With the last month
c) Within the last year
d) Most years
e) None

10) **If you circled/marked a, b, c or d to question above:**

a. Approximate average number of feral pigs per year (seen within 1 mile or less of your land/pigs) # ______________
b. What seasons do you typically see feral pigs within 1 mile or less of your land/pigs (Mark all that apply)
   i. Winter
   ii. Fall
   iii. Summer
   iv. Spring
c. Did you see more feral pigs in (choose one)
   i. drought years (2015/2016)
   ii. wet years (2017)
   iii. haven't seen a difference in total numbers or presence

11) **What was the estimated distance between the feral pig(s) seen and your domestic pigs:** (Choose the best answer)

a. Feral pig(s) had direct contact with my outdoor pig(s)
b. Within 100 ft. or less
c. Within 500 ft. or less
d. Within 1 mile or less
e. Within 2 miles or less
f. More than 2 miles away
g. NA

12) Have feral pigs been seen in fields or pastures or areas used for your outdoor raised pigs in the past 2 years?

- Yes
- No
- NA- no feral pigs nearby

13) Have you seen evidence of direct contact (within 10 ft) between feral pigs and your pig(s) in the past 2 years? (Mark all that apply)

- Yes
- Not direct contact
- No feral pigs nearby

14) Have you seen evidence of feral pigs mating with your domestic swine?

- Yes
- No
- NA- no feral pigs nearby

15) Have you seen evidence of feral pigs using/sharing the feeders of your domestic swine?

- Yes
- No
- NA- no feral pigs nearby

16) Do you consider feral pigs to be a nuisance for your livestock or land?

- Yes
- No
- NA: No feral pigs nearby

17) Do feral pigs bring any advantages to your operation and/or landscape?

- Yes
- No
- NA: No feral pigs nearby

18) Do you or other people hunt feral pigs in your county?

If yes, briefly describe where (if known)

____

19) Do you use any of the following to control feral pigs in the past 2 years? (Mark all that apply)

a. Traps
b. Dogs
c. Poison bait
d. Depredation
e. Hunting
    iv. During the day
    v. During the night
f. Fencing: (Standard or electric fencing, etc.)
g. Other _____________
h. None or no feral pigs nearby

20) Approximately what was the total number of acres (that you own, lease or manage) that was negatively impacted (i.e. damaged irrigation pipes, contaminated or consumed field of melons, etc.) by feral pigs in 2016 or 2017?

a. Total # acres (on average per year)_____________
b. NA- feral pigs are near my land/pigs but we have no feral pig damage
c. NA- no feral pigs nearby

21) Has there been feral pig damage to your livestock or land within the past: (Choose the best, most recent answer)

a. 1 year or less
b. 2-3 years
c. 4-6 years
d. 7+ Years
e. NA- feral pigs are near my land/pigs but we have no feral pig damage
f. NA- no feral pigs nearby

22) Which of the following areas/items on your farm/ranch/land did feral pigs damage during 2016 or 2017? (Mark all that apply)

a. Commercial crops for sale (vegetables, flowers, grains, etc.)
b. Home garden
c. Orchards
d. Vineyards
e. Forests
f. Wetlands or other surface water sources (creeks, rivers, ponds, etc.)
g. Fences or buildings
h. Irrigation systems (wells, pumps, drip lines, etc.)
i. Livestock (direct contact or injury, etc.)
j. Pastures
k. Stored livestock feed (hay, grain, etc.)
l. Other: ___________________________

23) Whether you own, lease or manage this land or items damaged by feral pigs in 2016 or 2017, what is your estimated dollar amount of damage from feral pigs?

- Total approximate $ per year on average: $___________
- NA- no damage from feral pigs

C. Demographics

24) How many acres do you use to raise pigs (on average per year) for the past 2 years?

a. Total # acres of farm/backyard/operation ________________
b. Total # of acres used for raising pigs________________

25) How many years have you raised pigs? (Choose the best answer)

c. 0-4
d. 5-9
e. 10 years or more

26) What CA county do your domestic pigs reside? ______________________________

27) The land where I raise my pig(s) is located: (Mark all that apply)

f. In a rural environment (with other farms, ranches, etc.)
g. In or near (within 2 miles) of wild lands (forest, BLM land, national park, etc.)
h. In or near (within 2 miles) suburbs
i. In or near (within 2 miles) of a town
j. In or near (within 2 miles) of a city

28) By providing us with the address of the location where you raise your domestic pigs (or <u>at a minimum the two nearest cross streets, city and zip code), you will be helping University of California Cooperative Extension determine</u> regions of risk for outdoor-raised pigs interfacing with feral pigs and identify key areas of California for control and/or surveillance of feral pig populations.

Address line 1:

City

Zip code

OR:

Nearest 1st cross street:

Nearest 2nd cross street:

City

Zip code

D. Domestic Swine Health

29) Have you seen any of these signs/symptoms in your outdoor-based pig herd in the past 2 years: (Mark all that apply)

k. Lameness
l. Respiratory signs (sneezing, coughing ("barking"), respiratory distress, pneumonia, nasal discharge, etc.)
m. Gastrointestinal signs (diarrhea, etc.)
n. Blisters, sore mouth, not eating
o. High mortality in piglets (over 5-10%)
p. Sloughing toe nails
q. Not eating
r. None of these listed above

Thank you for completing this voluntary, confidential survey for University of California Cooperative Extension (UCCE)!

Please mail completed surveys to:

Dr. Alda Pires
UC Davis
VM3B: Office 4209B
1089 Veterinary Medicine Drive
Davis, CA 95616

For questions contact Dr. Alda Pires:

apires@ucdavis.edu
Phone: 530-754-9855
