# Supplementary figures and images for: Identification of high-risk contact areas between feral pigs and outdoor-raised pig operations in California: Implications for disease transmission in the wildlife-livestock interface
Source: PLoS One. 2022 Jun 28;17(6):e0270500. doi: 10.1371/journal.pone.0270500 (PMC9239460; doi:10.1371/journal.pone.0270500)

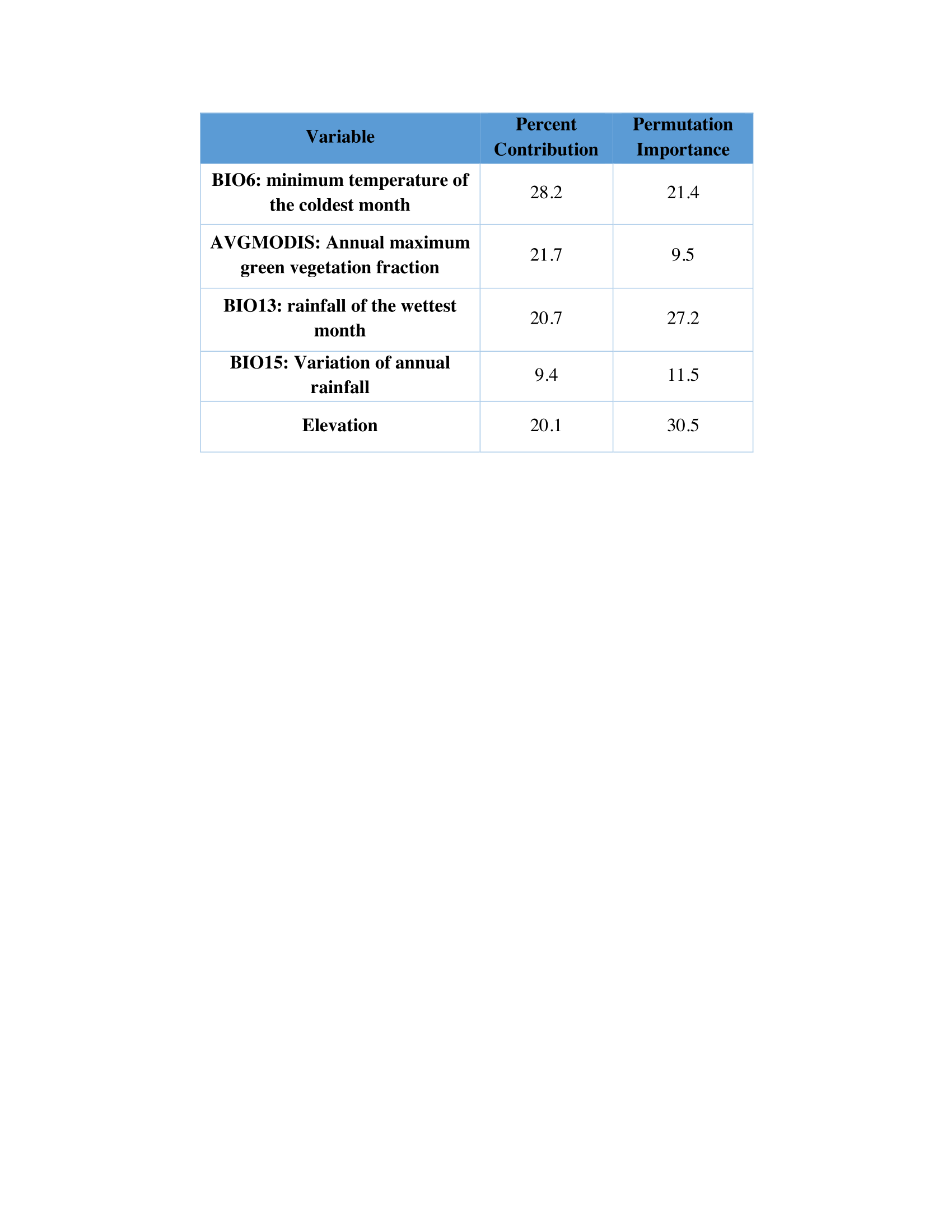

Supplement: S1 Table — (TIF) [file pone.0270500.s002.tif]

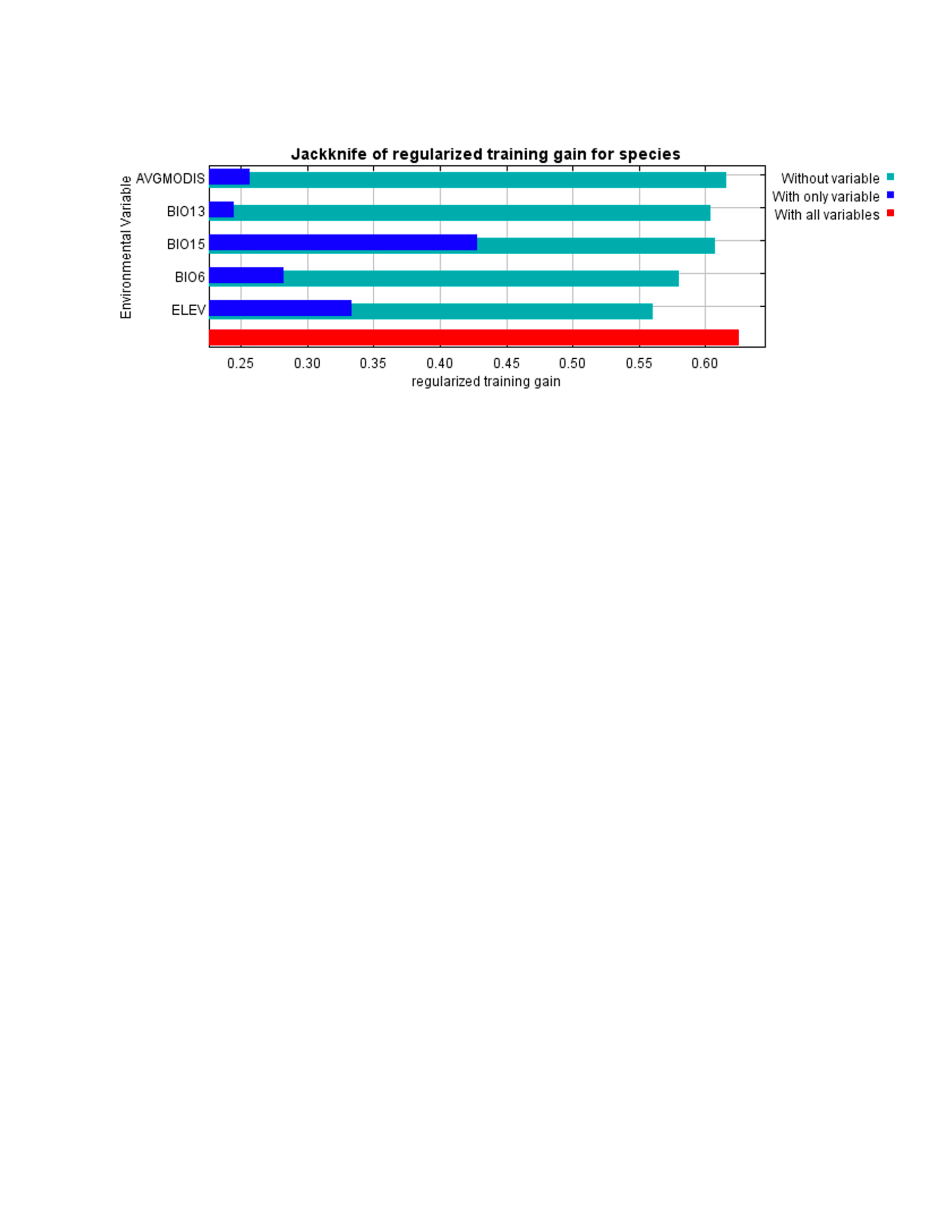

Supplement: S1 Fig — The Jackknife graph indicated importance of key variables: BIO6 was the minimum temperature of the coldest month, AVGMODIS was the annual maximum green vegetation fraction, BIO13 was the precipitation of the wettest month, BIO15 was the variation of annual precipitation and elevation. (TIF) [file pone.0270500.s003.tif]
