# Supplementary material for: Identification of high-risk contact areas between feral pigs and outdoor-raised pig operations in California: Implications for disease transmission in the wildlife-livestock interface
Source: PLoS One. 2022 Jun 28;17(6):e0270500. doi: 10.1371/journal.pone.0270500 (PMC9239460; doi:10.1371/journal.pone.0270500)
Supplement: S1 File — (ZIP) [file pone.0270500.s004.zip › Data Files 2022/Data Files Legends.docx]

**Data Files**

**April 2022**

**MaxEnt Manuscript**

**Supplementary data files:**

- AVGMODIS 2001-2012 Annual maximum green vegetation fraction (percent): 12 years of normalized difference vegetation index data
- BIO6 1970-2000 minimum ºC temperature of the coldest month
- BIO13 1970-2000 precipitation of the wettest month (mm)
- BIO15 1970-2000 coefficient of variation for seasonal precipitation (percent)
- ELEVATION (meters)
- 2017 Feral Pig Hunting Tags
  - Latitude
  - Longitude
  - CoName = County Name
  - MapsiteKC= specific site details
  - Year
  - Harvest date= date of collected feral pig
  - Count = if more than one
