# Supplementary material for: Identification of high-risk contact areas between feral pigs and outdoor-raised pig operations in California: Implications for disease transmission in the wildlife-livestock interface
Source: PLoS One. 2022 Jun 28;17(6):e0270500. doi: 10.1371/journal.pone.0270500 (PMC9239460; doi:10.1371/journal.pone.0270500)

PLOS ONE

Identification of high-risk contact areas between feral pigs and outdoor-raised pig operations in California: implications for disease transmission in the wildlife-livestock interface

--Manuscript Draft--

Manuscript Number:	PONE-D-21-36324
Article Type:	Research Article
Full Title:	Identification of high-risk contact areas between feral pigs and outdoor-raised pig operations in California: implications for disease transmission in the wildlife-livestock interface
Short Title:	Regions at high-risk in California for contact between feral pig and outdoor-raised domestic pigs
Corresponding Author:	Alda F A Pires, DVM, MPVM, PhD, DACVPM<br>University of California Davis<br>Davis, CA UNITED STATES
Keywords:	Maximum Entropy; pasture-based; swine, prediction; mapping; wild hogs
Abstract:	T<br>The US is currently experiencing a return to raising domestic pigs outdoors, due to consumer demand for sustainably-raised animal products. A challenge in raising pigs outdoors is the possibility of these animals interacting with feral pigs and an associated risk of pathogen transmission. California has one of the largest and widest geographic distributions of feral pigs. Locations at greatest risk for increased contact between both swine populations are those regions that contain feral pig suitable habitat located near outdoor-raised domestic pigs.<br>The main aim of this study entailed identifying potential high-risk areas of disease transmission between these two swine populations. Aims were achieved by predicting suitable feral pig habitat using Maximum Entropy (MaxEnt); mapping the spatial distribution of outdoor-raised pig operations (OPO); and identifying high-risk regions where there is overlap between feral pig suitable habitat and OPO.<br>A MaxEnt prediction map with estimates of the relative probability of suitable feral pig habitat was built. Predictor layers were included in variable selection steps for model building. For the risk map, the final MaxEnt model was overlapped with the location of OPO to categorize areas at greatest risk for disease transmission. Five variables were identified as important in predicting suitable feral pig habitat in the final model including the annual maximum green vegetation fraction, elevation, the minimum temperature of the coldest month, precipitation of the wettest month and the coefficient of variation for seasonal precipitation. The risk map reflects areas at greatest risk for contact between feral swine and domestic pigs raised outdoors and subsequent potential disease transmission.<br>Since raising pigs outdoors is a remerging trend, feral pig numbers are increasing nationwide, and both groups are reservoirs for various pathogens, the contact between these two swine populations has important implications for disease transmission in the wildlife-livestock interface.
Order of Authors:	Laura Patterson, PhD
	Jaber Belkhiria, DVM, MPVM, PhD
	Beatriz Martínez-López, DVM, MPVM, PhD
	Alda F A Pires, DVM, MPVM, PhD, DACVPM
Additional Information:	
Question	Response
Financial Disclosure<br><br>Enter a financial disclosure statement that	This study was supported by the Agriculture and Food Research Initiative grant no. 2019-67011-29609 / project accession no. 1019249 from the USDA National Institute of Food and Agriculture and a University of California, Davis Academic Federation

Powered by Editorial Manager® and ProduXion Manager® from Aries Systems Corporation

describes the sources of funding for the work included in this submission. Review the submission guidelines for detailed requirements. View published research articles from *PLOS ONE* for specific examples.<br><br>This statement is required for submission and **will appear in the published article** if the submission is accepted. Please make sure it is accurate.<br><br>**Unfunded studies**<br>Enter: *The author(s) received no specific funding for this work.*<br><br>**Funded studies**<br>Enter a statement with the following details:<br>• Initials of the authors who received each award<br>• Grant numbers awarded to each author<br>• The full name of each funder<br>• URL of each funder website<br>• Did the sponsors or funders play any role in the study design, data collection and analysis, decision to publish, or preparation of the manuscript?<br>• **NO** - Include this sentence at the end of your statement: *The funders had no role in study design, data collection and analysis, decision to publish, or preparation of the manuscript.*<br>• **YES** - Specify the role(s) played.<br><br>* typeset	grant.<br>The funders had no role in study design, data collection and analysis, decision to publish, or preparation of the manuscript.
**Competing Interests**<br><br>Use the instructions below to enter a competing interest statement for this submission. On behalf of all authors, disclose any competing interests that could be perceived to bias this work—acknowledging all financial support and any other relevant financial or non-financial competing interests.<br><br>This statement is required for submission and **will appear in the published article** if the submission is accepted. Please make sure it is accurate and that any funding	No competing interests or conflict of interests

sources listed in your Funding Information later in the submission form are also declared in your Financial Disclosure statement.<br><br>View published research articles from *PLOS ONE* for specific examples.<br><br>**NO authors have competing interests**<br><br>Enter: *The authors have declared that no competing interests exist.*<br><br>**Authors with competing interests**<br><br>Enter competing interest details beginning with this statement:<br><br>*I have read the journal's policy and the authors of this manuscript have the following competing interests: [insert competing interests here]*<br><br>* typeset	
**Ethics Statement**<br><br>Enter an ethics statement for this submission. This statement is required if the study involved:<br><br>• Human participants<br>• Human specimens or tissue<br>• Vertebrate animals or cephalopods<br>• Vertebrate embryos or tissues<br>• Field research<br><br>Write "N/A" if the submission does not require an ethics statement.<br><br>General guidance is provided below. Consult the submission guidelines for detailed instructions. **Make sure that all information entered here is included in the Methods section of the manuscript.**	This study used secondary data, doesn't included field work, nor animal primary data. An IRB protocol was reviewed and determined to be exempted by the UC Davis IRB. See Manuscript lines 180-183: The survey instrument and protocols were reviewed and exempted by the Institutional Review Board (IRB) of the University of California-Davis (No. 1180798-1).

Powered by Editorial Manager® and ProduXion Manager® from Aries Systems Corporation

**Format for specific study types**<br><br>**Human Subject Research (involving human participants and/or tissue)**<br>• Give the name of the institutional review board or ethics committee that approved the study<br>• Include the approval number and/or a statement indicating approval of this research<br>• Indicate the form of consent obtained (written/oral) or the reason that consent was not obtained (e.g. the data were analyzed anonymously)<br><br>**Animal Research (involving vertebrate animals, embryos or tissues)**<br>• Provide the name of the Institutional Animal Care and Use Committee (IACUC) or other relevant ethics board that reviewed the study protocol, and indicate whether they approved this research or granted a formal waiver of ethical approval<br>• Include an approval number if one was obtained<br>• If the study involved *non-human primates*, add *additional details* about animal welfare and steps taken to ameliorate suffering<br>• If anesthesia, euthanasia, or any kind of animal sacrifice is part of the study, include briefly which substances and/or methods were applied<br><br>**Field Research**<br><br>Include the following details if this study involves the collection of plant, animal, or other materials from a natural setting:<br>• Field permit number<br>• Name of the institution or relevant body that granted permission	
**Data Availability**<br><br>Authors are required to make all data underlying the findings described fully available, without restriction, and from the time of publication. PLOS allows rare exceptions to address legal and ethical concerns. See the PLOS Data Policy and FAQ for detailed information.	No - some restrictions will apply

A Data Availability Statement describing where the data can be found is required at submission. Your answers to this question constitute the Data Availability Statement and **will be published in the article**, if accepted.<br><br>**Important:** Stating 'data available on request from the author' is not sufficient. If your data are only available upon request, select 'No' for the first question and explain your exceptional situation in the text box.<br><br>Do the authors confirm that all data underlying the findings described in their manuscript are fully available without restriction?	
**Describe where the data may be found in full sentences. If you are copying our sample text, replace any instances of XXX with the appropriate details.**<br><br>• If the data are **held or will be held in a public repository**, include URLs, accession numbers or DOIs. If this information will only be available after acceptance, indicate this by ticking the box below. For example: *All XXX files are available from the XXX database (accession number(s) XXX, XXX.).*<br>• If the data are all contained **within the manuscript and/or Supporting Information files**, enter the following: *All relevant data are within the manuscript and its Supporting Information files.*<br>• If neither of these applies but you are able to provide **details of access elsewhere**, with or without limitations, please do so. For example:<br><br>*Data cannot be shared publicly because of [XXX]. Data are available from the XXX Institutional Data Access / Ethics Committee (contact via XXX) for researchers who meet the criteria for access to confidential data.*<br><br>*The data underlying the results presented in the study are available from (include the name of the third party*	OPO data cannot be shared publicly, as they contain GPS coordinates and participants provided locations based on confidentiality. Feral pig hunting tags may be available through CDFW. Predictor layers used to build a MaxEnt model are publicly available online.

and contact information or URL).<br>• This text is appropriate if the data are owned by a third party and authors do not have permission to share the data.<br><br>* typeset	
Additional data availability information:	

Powered by Editorial Manager® and ProduXion Manager® from Aries Systems Corporation

Manuscript

Click here to access/download;Manuscript;MaxEnt Risk Map FINAL PlosOne v2.docx

Identification of high-risk contact areas between feral pigs and outdoor-raised pig operations in California: implications for disease transmission in the wildlife-livestock interface

Laura Patterson[1,2], Jaber Belkhiria[2], Beatriz Martínez-López[2], Alda F. A. Pires[1*]

[1] Department of Population Health and Reproduction, University of California-Davis, Davis, California, United States of America

[2] Center for Animal Disease Modeling and Surveillance (CADMS), University of California-Davis, Davis, California, United States of America

*Corresponding author

E-mail: apires@ucdavis.edu (AP)

Short title: Regions at high-risk in California for contact between feral pig and outdoor-raised domestic pigs

Abstract

The US is currently experiencing a return to raising domestic pigs outdoors, due to consumer demand for sustainably-raised animal products. A challenge in raising pigs outdoors is the possibility of these animals interacting with feral pigs and an associated risk of pathogen transmission. California has one of the largest and widest geographic distributions of feral pigs. Locations at greatest risk for increased contact between both swine populations are those regions that contain feral pig suitable habitat located near outdoor-raised domestic pigs.

The main aim of this study entailed identifying potential high-risk areas of disease transmission between these two swine populations. Aims were achieved by predicting suitable feral pig habitat using Maximum Entropy (MaxEnt); mapping the spatial distribution of outdoor-raised pig operations (OPO); and identifying high-risk regions where there is overlap between feral pig suitable habitat and OPO.

A MaxEnt prediction map with estimates of the relative probability of suitable feral pig habitat using hunting tags as presence-only was built. Predictor layers were included in variable selection steps for model building. For the risk map, the final MaxEnt model was overlapped with the location of OPO to categorize areas at greatest risk for disease transmission. Five variables were identified as important in predicting suitable feral pig habitat in the final model including the annual maximum green vegetation fraction, elevation, the minimum temperature of the coldest month, precipitation of the wettest month and the coefficient of variation for seasonal precipitation. The risk map reflects areas at greatest risk for contact between feral swine and domestic pigs raised outdoors and subsequent potential disease transmission.

Since raising pigs outdoors is a remerging trend, feral pig numbers are increasing nationwide, and both groups are reservoirs for various pathogens, the contact between these two

swine populations has important implications for disease transmission in the wildlife-livestock interface.

Introduction

Although a majority of commercial swine production in the United States (US) occurs indoors with high levels of biosecurity, the US is currently experiencing a return to raising domestic pigs outdoors [1,2]. Before the 1950s, most swine operations in the US were small-scale family farms and either a hybrid of indoor/outdoor or solely outdoor-based [1,3]. Beginning in the 1960s, commercial swine production began transitioning to indoor systems, based on goals to increase efficiency and reduce swine disease transmission (e.g., brucellosis) as well as a public health mandate to decrease human trichinosis cases [4-7]. However, consumer demand for sustainable or pasture-raised animal products within the past few decades has revived traditional methods of raising swine outdoors or on pasture (i.e., outdoor-raised pigs, pasture-based) [1,2,8,9]. While primarily considered a niche production method in the US, outdoor-raised pig operations (OPO) (e.g., commercial pork producers, backyard operations) are broadly distributed throughout California.

A challenge in raising pigs outdoors is the possibility of these animals interacting with wildlife disease reservoirs, such as feral pigs, and an associated risk of zoonotic and/or swine pathogen transmission [10-16]. Both domestic and feral pigs share the same genus and species (*Sus scrofa*) and can be reservoirs for zoonotic pathogens (e.g., swine influenza virus, Shiga toxin-producing *Escherichia coli*) [17-23]. Also, swine diseases eradicated in conventional indoor-raised herds (e.g., pseudorabies, brucellosis) have been documented in feral swine in California and contact between feral pigs and outdoor-raised swine herds is a risk factor for the reintroduction of these diseases to domestic herds in the US [6,7,15,19,24-26]. For example, a

2016 human case of brucellosis in New York state was traced to a feral pig intrusion event on a pasture-raised pig farm. *Brucella suis* was then transmitted to domestic pigs raised outdoors in 13 other states through animal sales [25,27]. Feral pigs could also play a significant role in the transmission and maintenance of transboundary animal diseases (TAD) introduced to North America [11,16,28,29]. For instance, African Swine Fever (ASF) is actively spreading in eastern Europe, with wild boars transmitting this devastating disease between and within countries [30]. Similarly, wild boars abet the transmission of ASF in South Korea, spreading the virus to outdoor-raised swine [31,32]. And most recently, ASF was identified in domestic swine in the Dominican Republic, which is the closest to the US that ASF as spread in this century [33].

During the past few decades, feral pig populations have greatly increased in the US from 17 to 41 states [34-36]. California has one of the largest and widest geographic distributions of feral pigs and this invasive species has the broadest habitat range of any large mammal except humans, which is in part due to their ability to adapt to a diverse range of ecological habitats and their opportunistic omnivore diet [36-41]. Feral pig population distribution and abundance is dynamic yet has not been documented at fine spatial units. Previous presence maps reported feral pigs for an entire county, even if there had only been a single occurrence recorded countywide [38,42,43].

Hypothetically, an area is at higher risk of disease transmission if it is more likely to experience interactions between feral pig and domestic pigs raised outdoors, as these outdoor-based pigs can serve as a conduit for disease spread from wildlife to humans. Locations at greatest risk for increased contact between both swine populations are those regions that contain feral pig suitable habitat located near outdoor-raised domestic pig premises, especially those OPO with relatively low levels of biosecurity [24,26,36,44]. Contact between feral pigs and

outdoor-raised pigs in California has been documented, as feral pigs are attracted to agricultural regions for food, water and mates [10,12,29,45-48]. There is enormous value in identifying agricultural regions with a higher probability of feral pig contact, because these areas could benefit from targeted cost-effective disease surveillance and risk-mitigation strategies to prevent disease transmission.

Predicting suitable habitat for feral pigs (i.e., likelihood of feral pig presence) in combination with spatially characterizing the distribution of OPO can provide an important tool to ascertain possible high-risk areas of contact at the feral-domestic pig interface and identify future disease spillover areas [47,49,50]. Species distribution modeling (SDM) methods have been widely used in ecological studies and are becoming popular for use in epidemiological investigations of disease transmission between wildlife and livestock [48,51-53]. Maximum Entropy (MaxEnt), which is one type of SDM, allows usage of presence-only data for the species of interest (i.e., feral pigs) [54]. In combination with biologically-appropriate covariate factors, MaxEnt is able to spatially predict the probability of suitable habitat for a species for a chosen spatial unit (i.e., pixel) [55].

These two parallel trends of expanding feral pig populations and a resurgence of raising domestic swine outside has important implications for disease transmission, which could negatively impact both public health and California's agricultural industry. To the best of our knowledge, there are no maps characterizing where suitable feral pig habitat overlaps with domestic pigs raised outdoors at the farm-level in California. The overall objective of this study entailed spatially identifying potential high-risk areas of disease transmission between these two swine populations. This objective was achieved by a three-step process: 1) predicting suitable feral pig habitat in California using MaxEnt; 2) mapping the spatial distribution of OPO in

California; and 3) identifying high-risk regions where there is spatial overlap between feral pig suitable habitat and OPO, as potential disease transmission areas.

Materials and methods

Maximum Entropy model

MaxEnt is an established SDM method that produces an output prediction map containing estimates of the relative probability of suitable habitat areas for the species of interest (i.e., feral pigs) within each pixel, using presence-only points and predictors (i.e., covariate spatial layers) [32,46,50,54,56-60]. For feral pig presence data, we obtained feral pig hunting tags from 2012-19 that were cleaned and recorded with GPS coordinates by the California Department of Fish and Wildlife (CDFW). Hunters in California are voluntarily asked to report feral pig harvest locations by submitting hunting tags to CDFW. Using hunting records for presence-points of feral pigs or wild boars has been used in previous studies [48,61]. CDFW 2012-19 feral pig hunting tags totaled 5,148 after removing duplicates. Due to the large amount of data points, hunting tags were also manually filtered (i.e., subsampled) by year as a way to decrease the abundance of points before running models to reduce sampling bias and increase model stability, as suggested by previous analyses of MaxEnt [48,60,62-64].

Publicly available predictor spatial layers online, including biotic (e.g., land cover, vegetation) and abiotic (e.g., temperature, precipitation, elevation), were included in variable selection steps, and were chosen based on known feral pig behaviors, habitat and food preferences (Table 1) [37,46,65-69]. For example, AVGMODIS was the annual maximum green vegetation fraction (MGVF) combined with 12 years of normalized difference vegetation index data (NDIV) and relates to food and shrub cover for feral pigs [70-72]. Other variables included elevation, as feral pigs may prefer specific altitudes, and nineteen environmental variables from

the WorldClim set of 30 year trend climatic factors [73]. Examples of environmental variables used from the WorldClim site included the minimum temperature of the coldest month (BIO6), precipitation of the wettest month (BIO13) and the coefficient of variation for seasonal precipitation (BIO15) [74]. Layers were reprojected to the Albers Equal-Area coordinate reference system for California ("California Albers" (meters)) using QGIS 3.6 [75]. The resolution size that used a reasonable amount of computer computation time, while maintaining adequate detail for suitable habitat modeling, was 270m x 270m.

Table 1: Publicly available predictor layers assessed during variable selection for Maximum Entropy model building.

Name	Description	Source
**AVGMODIS***	Annual maximum green vegetation fraction: 12 years of normalized difference vegetation index data	modis.gsfc.nasa.gov/data/
**CropScape**	USDA National Agricultural Statistics Service Cropland Data	https://nassgeodata.gmu.edu/CropScape/
**ELEVATION***	Elevation/Altitude	www.worldclim.org/
**FVEG**	Statewide vegetation with WHR types, size and density.	https://frap.fire.ca.gov/mapping/gis-data/
**GAP**	USGS GAP analysis project: land cover	https://www.usgs.gov/core-science-systems/science-analytics-and-synthesis/gap/science/
**Global Human Influence Index**	Nine global data layers: human population pressure, human land use and infrastructure, and human access	https://sedac.ciesin.columbia.edu/data/set/wildareas-v2-human-influence-index-geographic/maps
**NDVI**	Normalized difference vegetative index	https://data.nodc.noaa.gov/cgi-bin/iso?id=gov.noaa.ncdc:C00813
**NLCD**	National Land Cover Database	https://www.mrlc.gov/data
**Open Water**	Multiple integrated global remote sensing-derived land-cover products and prevalence of 12 land-cover classes	http://www.earthenv.org/

**PRISM**	Seven climatic variables for the US annual and monthly precipitation and temperature	http://www.prism.oregonstate.edu/
**Streams**	Streams in the US (distance to water)	https://catalog.data.gov/dataset/cdfg-100k-streams-2003
**USDA zones**	Hardiness zones based on mean extreme annual minimum temperatures	https://planthardiness.ars.usda.gov/
**WorldClim***	19 Bioclimatic variables: 30-year averages 1970-2000	www.worldclim.org/bioclim

*Indicates variables included in the final MaxEnt model.

MaxEnt models were built in R Statistical Software version 0.98.110253 © [76]. The following R packages were used to run MaxEnt: dismo, sp, and raster [57,77-80]. MaxEnt settings were chosen based on previously published literature and included using 25 random test points, 15 replicates, 5000 maximum iterations and the 10-percentile training for the threshold rule [51,53,55,57,81]. A regularization multiplier of 1 through 5 was assessed to avoid overfitting and the default 1 was determined to be the optimal setting for the final model [81]. Logistic values for output was used as well as cross validation, which separates presence points into 80% training and 20% testing data (i.e., model validation), using k-fold sub-sampling to fit a model [51,55,82]. The relative contribution of each variable in a MaxEnt model was assessed comparing both percent contribution and permutation of importance, averaged over the number of iterations run and ascertained by jackknife tests [51,54,82]. Predictors for the final model were assessed using a backward variable selection approach: variables remained at each step if their percent contribution or permutation importance was approximately 10% or more [32,51]. The response curves generated within MaxEnt showed the predicted probability of suitable feral pig habitat for each individual variable, changing per each level of the predictor [65,68,83].

MaxEnt model performance was assessed using the area under the curve (AUC) of the receiver operator characteristic (ROC), averaged over the number of chosen replicate runs [51,53,84]. AUC reflects a model's prediction ability, on a scale of 0 to 1.00, with 0.50 representing random chance. While AUC is a standard diagnostic method to evaluate MaxEnt models, some authors suggest calibrating the AUC (i.e., AUCc), which removes spatial sorting bias (ssb) (i.e., spatial autocorrelation) by using point-wise distance sampling [57,77,85]. A ssb close to 1 indicates no spatial sorting bias, whereas a ssb close to 0 suggests a large spatial bias, and the need to use AUCc [85]. The final model was chosen based on the highest AUCc, relative to other models.

Risk map and OPO

The feral pig-domestic pig risk map was built by overlapping California OPO locations with the final MaxEnt feral pig suitable habitat raster. Between 2014-2019, a list of California OPO was compiled through various sources (e.g., agricultural festivals, local farmers markets, University of California Cooperative Extension (UCCE) advisors, web-based searches (search terms: "pasture-raised pork", "pastured pigs"). GPS coordinates for all OPO were identified using Google Earth Pro v7.3.3 [86]. Additionally, an online survey that contained an interactive map component was built with Survey 123 v3.6 [87]. The survey contained 29 questions that consisted mainly of multiple choice questions, with a few open ended questions about the number of animals raised (e.g., how many sows or boars raised on average each year). The survey included questions regarding biosecurity practices, swine health and feral pig presence. This online survey was announced electronically (e.g., media, e-newsletters) to swine related groups and organizations or conducted in-person at events, such as agricultural fairs. The survey instrument and protocols were reviewed and exempted by the Institutional Review Board (IRB)

of the University of California-Davis (No. 1180798-1).

To build a risk map for California, the final MaxEnt model predicting suitable habitat for feral pigs was overlapped with the location of OPO to categorize areas at greatest risk for disease transmission, due to contact between these two swine populations, and characterize risk at the farm-level. The underlying assumption presumed that direct or indirect contact between feral pigs and domestic pigs raised outdoors is a risk for disease transmission. The probability of suitable habitat for feral pigs was extracted from the final MaxEnt model for each OPO location, using the Sample Raster Value tool in QGIS and added to the OPO shapefile. Then the Kernel Density tool in QGIS was used to make the risk map, matching the 270m x 270m resolution of the MaxEnt model and using the MaxEnt model probabilities as weights. Additionally, we used a radius of 5 km at each OPO location, which was an extrapolated average estimate from US based studies that measured home range of feral pigs, understanding that home ranges vary depending on age and gender of animal, as well as resource availability [46,67,88,89]. The Kernel Density map was overlaid with the final MaxEnt model.

Results

MaxEnt model

The final MaxEnt model was chosen based on the highest AUCc of 89.7, relative to other models. Probability values for suitable habitat were divided into five equal interval categories: minimal (< 0.01); low (0.01-0.22); moderate (0.23-0.43); high (0.44-0.65); and extremely high (0.66-0.87), with 0.87 being the highest predicted probability in the final MaxEnt model (Fig 1). Areas with the highest likelihood of suitable feral pig habitat in California (i.e., orange, and red categories) included the north coast from Mendocino County all the way south along the coast to Santa Barbara County, and counties that border these coastal counties (e.g., Lake, Napa, Contra

Coast, Santa Clara and San Benito). Additionally, suitable habitat areas included the foothills of the Sierra mountains, from Shasta County south to Tulare County. Least likely suitable habitat included the Central Valley and eastern counties of California, from the most northern county of Modoc all the way to Imperial County in the south.

Fig 1: Final MaxEnt model predicting suitable feral pig habitat in California. Color-coded categories represent the probability of suitable feral pig habit on a scale of almost zero (<0.01) to extremely high (0.66-0.87), based on equal intervals.

Five variables were identified as significant in predicting suitable feral pig habitat. The five significant variables were the annual maximum green vegetation fraction (AVGMODIS), the minimum temperature of the coldest month (BIO6), precipitation of the wettest month (BIO13) and the coefficient of variation for seasonal precipitation (BIO15) and elevation. All five variables provided approximately 10% or more percent contribution and permutation importance to the final model. (S1 Table). The jackknife test results provided more information regarding the importance of each variable in the final model (S1 Fig). For example, BIO15 was the variable with the highest gain when used alone and elevation had the most information that was not available in the other variables. The response curves for the significant five variables indicated the predicted suitability range of each variable for feral pigs (i.e., the x-axis values above 0.50 on the y-axis) (S2 Fig). For instance, feral pigs are predicted to prefer vegetative cover (i.e., AVGMODIS) of at least 60% or more.

Risk map and OPO

A total of 305 OPO were identified between 2014-2019, from 79.30% (46/58) of California's 58 counties (i.e., no OPO data for 12 counties). The most OPO were identified in the following counties: Sonoma (n=48), Mendocino (n=19), Nevada (n=16) and Yolo (n=12). From the online survey, 39 OPO locations were gathered from 44 respondents and included in the final total. All survey respondents raised domestic swine outdoors and 25.00% (11/44) had seen feral pig presence within 3.22 km or less of their domestic swine raised outdoors, with 15.91% (7/44) observing feral pigs within 152.4 m of their pig herd. Domestic pig herd size ranged from 1- 350 animals, with a mean of 24 and median of six. Hectares (ha) dedicated to raising pigs ranged from 0.026 ha to 12.14 ha with a mean of 1.89 ha and median of two 0.81 ha, with nine not answering.

The risk map reflects areas at greatest risk for contact between feral swine and domestic pigs raised outdoor and subsequent potential disease transmission (Fig 2). Risk levels start at green for low-risk areas and range up to orange and red for the highest risk areas. Areas with the most risk for contact between these two swine populations are denoted in orange or red, with sharper colors representing denser clustering of OPO. The counties with the highest likelihood of suitable feral pig habitat and densest clustering of OPO included: Sonoma, Marin, Napa, Yolo, Nevada, Mendocino and Lake counties. Areas at lowest risk include the full eastern edge of California, which includes the Cascadian and Sierra Nevada Mountain ranges as well as deserts in the south. Table 2 categorizes the distribution of OPO at each level of probable suitable feral pig habitat using the final MaxEnt model levels. The results indicated that 49.18% of the 305 OPO were located near extremely high or highly suitable feral pig habitat.

Fig 2: Risk map demonstrating areas in California at greatest risk for contact between feral pigs and outdoor-raised domestic pigs within a 5km radius from each farm, using the Kernel Density tool in QGIS. Colors are based on the probability of suitable feral pig habitat from the final MaxEnt model at each OPO, with sharper colors representing denser clustering of OPO.

Table 2: Percentage of 305 OPO identified in each MaxEnt suitable feral pig habitat level. The final MaxEnt model contains a probability scale of 0.00 to 0.87 and was divided into equal intervals.

Levels	%OPO (ct/305)
**Minimal (< 0.01)**	0.98% (3/305)
**Low (0.01-0.22)**	19.67% (60/305)
**Moderate (0.23-0.43)**	30.16% (92/305)
**High (0.44-0.65)**	25.90% (79/305)
**Extremely high (0.65 +)**	23.28% (71/305)

Discussion

In this study, we built a feral pig suitable habitat prediction model for California using MaxEnt at a fine scale of 270m x270m. Significant predictors of suitable feral habitat included precipitation, minimum temperature, elevation, and percentage of vegetation. Additionally, this study overlapped MaxEnt predicted suitable feral pig habitat and outdoor-raised pig operations to create a risk map for potential disease transmission in California at the feral pig-domestic pig interface. To the best of our knowledge, this is one of the first studies that identified areas at risk for feral and domestic pig contact in California. Although previous studies discussed the possibility of feral pig populations spreading disease to outdoor-raised pigs at the county level, to our knowledge, this is the first study to predict risk at the farm-level in California.

Since the exact location of most feral pig populations is unknown, species distribution predictive methods like MaxEnt are important to understand where feral pigs could potentially interface with domestic swine raised outside, either currently or in the future. Our final MaxEnt prediction model provides a more informative picture of suitable habitat for feral pigs than previous studies, which only showed single presence points or reported feral pigs at the county level, even if only one feral pig was identified in that county [36,38,42,43]. For instance, although previous county-level maps indicated that all California counties except for Imperial County harbored feral pigs, our MaxEnt model shows almost no suitable habitat in an additional five counties: Modoc, Mono, Alpine, Lassen and Inyo. This result may indicate that few feral pigs have been seen in those counties.

Additionally, the final MaxEnt model was based on a fine spatial scale and indicated heterogenous suitable habitat, not a uniform distribution, for each county, which is compatible with the fact that feral pigs need shrub cover and food to survive, which would not be found in

cities or deserts [41]. Earlier feral pig mapping studies by the Southeastern Cooperative Wildlife Disease Study and National Feral Swine Program (NFSP) focused on the entire US and only county level occurrence of feral pigs [42,43,90]. A 2015 USDA study overlapped NFSP county-based feral pig locations with data from the 2012 NAHMS study of small-enterprise swine operations, specifically whether these survey respondents had seen feral pigs on their premises or within the same county, to ascertain the level of agreement between the two datasets [36]. They identified five counties in California that were in agreement with our MaxEnt model findings for suitable feral pig habitat: Mendocino, Tehama, Nevada, El Dorado, and San Luis Obispo, and two counties that differed: Ventura and Los Angeles counties. Our risk map reflects the heterogeneity of feral pig habitat in each county and identified high-risk contact areas between farms and feral pigs in California. Although these county-based maps are important to demonstrate the trend of increasing feral pig populations nationwide, stakeholders and feral pig disease surveillance agencies could benefit from targeting outreach and mitigation strategies to specific regions within a county using our maps.

The results of our final MaxEnt model indicated five variables that were useful in predicting suitable feral pig areas in California, including three WorldClim layers: the minimum temperature of the coldest month (BIO6), precipitation of the wettest month (BIO13) and the coefficient of variation for seasonal precipitation (BIO15). Other studies also used WorldClim factors to predict the distribution of wild boar or feral pigs. These bioclimatic variables have been widely used in environmental studies and are now becoming popular for use in epidemiological investigations [74]. These climate variables are 30 year averages and "capture broader biological trends better than the temperature or the amount of precipitation for a given day due to the inherent variability associated with weather" [74]. Bosch *et al* (2014) built a

MaxEnt model for wild boar in Spain and their model also contained BIO6 and BIO15 as did regional models built by Pittiglio *et al* (2018) with BIO13 being significant as well [69,91]. BIO6 is the minimum temperature of the coldest month and is interpreted as being a useful variable when deciding if the species of interest is affected by extreme cold events throughout a year [74]. Hill *et al* (2014) used MaxEnt to predict the distribution of *Trichinella* spp. and *Toxoplasma gondii* in feral pigs in the US and also identified BIO6 and elevation as significant predictor variables, along with land cover and other WorldClim factors [26]. The response curve for BIO6 in our model peaks at the predicted ideal range for feral pigs, with both ends indicating extreme cold temperatures that may be avoided by feral pigs. A 2015 study by McClure *et al* (2015) indicated that suitable feral pig habitat may be limited by cold temperatures, precipitation and water availability, which reflects our findings [46]. BIO13 is defined as precipitation of the wettest month and is useful if extreme rainfall patterns influence the range of feral pigs [74]. BIO15 measures the variation in annual precipitation totals per month (i.e., seasonality of precipitation) and reflects the variability of rainfall that may affect a species [74]. According to the Jackknife graph, the variable with the highest gain when used alone was BIO15, and therefore had the most important information for predicting suitable feral pig habitat. Snow *et al* (2017) used Bayesian methods to predict the expansion of feral pigs in the US, but also detected that temperature and precipitation levels were significant predictors [38].

The final MaxEnt model gain is decreased the most if elevation is ignored and therefore it has significant information that is not available from the other variables in predicting feral pig suitability. Elevation was also significant in the MaxEnt models built by Hill *et al* (2014) [26]. These results combined with the response curve possibly reflect feral pigs preference for lower altitudes in the US. AVGMODIS, a measure of the annual maximum green vegetation fraction

on a scale of 0 to 100, was also an important predictor of suitability, which reflects feral pigs' need for available food and vegetative cover [71]. Garza *et al* (2018) identified NDVI, which AVGMODIS is based upon, and precipitation as important variables in predicting home ranges of feral pigs or wild boar worldwide, using generalized linear models [65].

The significant layers identified in our study to predict feral pig suitability are not unique, and this may be due to the fact that feral pigs are a highly adaptable and opportunistic omnivores [38]. Lobe *et al* (2008) stated that MaxEnt AUC values will be lower for generalist species that are widely distributed [92]. However, the AUCc of our final model was 89.7, which indicates a good model. Additionally, 2017 hunting tags (n=1,745) vs. all 5,148 points for 2012-19 provided the best model. MaxEnt is an important method to predict the distribution of rare species, and an upper maximum range for the number of species occurrence points has not been previously determined. However, our result fits with a study conducted by Chen *et al* (2012) to determine the sample size for the outcome variable in building MaxEnt models [62]. They reported that standard deviation decreased and MaxEnt models became more stable using species occurrence points of 1,000-1,200 [62-64]. Most likely the sample size of the outcome variable that reaches asymptote is dependent on geographic extent and characteristics of the species of interest.

Regarding feral pig presence on farms, the most recent NAHMS survey asked participating swine small-enterprise producers in the US (i.e., those raising 100 pigs or less) about presence of feral swine in their county but did not separate farms based on whether they raised domestic swine indoors or outdoors. However, a 2015 United States Department of Agriculture (USDA) report regarding overlap of feral and domestic pigs in the US used this NAHMS dataset and reported that of 320 participating US counties, 74% of these counties had small-enterprise swine producers who allowed their pigs some level of outdoor access [36]. The

NAMHS results indicated that 52.9% of small-enterprise swine producers in the West/South region, which included California, reported feral pigs in the same county, with 16.2% of those having feral pig presence on their operation, similar to our survey results that showed 15.91% of respondents had seen feral pigs within 500 feet of their pig herd [93]. Another study that measured co-occurrence of feral pigs and agriculture to understand the risk of disease transmission, but did not separate outdoor versus indoor herds, reported that on average, 47.7% of all types of farms had feral pigs in the same counties, including California, showing a significant increase in the decade from 2002-2012 [94]. The results of these aforementioned survey-based studies indicated together that more than 45% of farms have feral presence within the same county, which matches the results from our risk map that showed almost half of the identified OPO had suitable feral pig habitat nearby [36,93,94]. These findings indicate the need for targeted outreach and mitigation strategies for those farms at highest-risk for feral pig contact, due to the potential for disease transmission between these two swine groups.

Studies that identified high-risk areas in California between feral pigs and domestic swine raised outdoors are sparse. A 2015 USDA report extracted outdoor operations with NFSP feral swine populations and did not identify any hot spots of overlap in California as seen in our results. However, they did not report the number of OPO per state or county and most likely our state-focused study identified more OPO than their survey-based national study. A study by Miller *et al* (2017) also assessed possible disease transmission between feral pigs and farm at the county level [94]. They reported that domestic swine, either raised indoors or outside, have been increasing in counties that also had feral pig presence. The lack of maps identifying areas at high-risk for disease transmission between these two swine populations indicates a need for further research. Additionally, the risk map identified eastern counties as having the lowest risk.

However, we did not identify OPO in many of these counties, therefore we cannot say there is no risk in these regions.

A limitation of this study involves using hunting tags as a proxy for presence of feral pigs to predict suitable habitat. Hunting tags are voluntarily submitted to CDFW by hunters and estimated to account for only 30% of all hunted pigs and most likely biased toward easy to access areas. Also, only half of the land in California is public land and accessible to hunters, therefore feral pigs hunted on private land are not included in our data sets. However, Rutten *et al* (2019) used similar hunting bags and MaxEnt to successfully predict the distribution of wild boar in Belgium [48]. And Alexander *et al* (2016) also used hunting records to predict wild boar habitat in Europe [61]. Additionally, MaxEnt assists in overcoming these challenges by identifying similar habitats in all parts of California and predicting suitable areas.

Both the MaxEnt model and risk map are limited because they are static maps that use fixed layers as their foundation; consequently, they do not incorporate dynamic events over various years (e.g., wildfires, landscape changes, weather fluctuations). Also, feral pigs may migrate seasonally due to shifting weather, resource availability, hunting pressure or wildfire and future research could focus on species distribution modeling that includes dynamic real time variables or remote sensing data; however, seasonal or dynamic spatial data are not available yet for most spatial predictors in California [46,48,65,88,95-97]. However, our approach is valuable as a first step in identifying multiple high-risk areas for future research, where additional data could be collected. Furthermore, future research could add feral pig disease data collected statewide to evaluate if high-risk areas for feral-domestic pig contact equates to those areas with higher prevalence of diseases [49].

There are some challenges and limitations to the risk map generated in this study. For instance, farms and ranches in California, including backyard and commercial operations, are not required to register with state agricultural agencies, therefore, the total number, distribution, and size of OPO remains unknown and are underrepresented in this study. A majority of the identified OPO in this study were commercial pork producers with an online presence or ones that attend conferences, farmers markets and fairs. If more OPO locations could be identified, than a more comprehensive map of high-risk areas could be generated. Additionally, because we are based at the University of California, Davis in Yolo County, there is selection bias in the OPO identified as our agricultural networks are within the UCCE network. Overrepresented counties reflected either sampling bias or clustering of these niche operations or both. Nevertheless, the number of OPO included in this study (n=305) and the fact that more than 40% of these operations were in highly suitable areas for feral pig contact is relevant as an initial approximation of a likely much larger risk of disease transmission at the feral-domestic swine interface in California. In the future, adding disease cases to this risk map would add additional epidemiological information regarding possible pathogen transmission.

Conclusion

This study evaluated the feral-domestic pig interface of two parallel trends: expanding feral pig populations and an increase in outdoor-raised pig operations in California, as related to the risk for future disease transmission. Since both swine populations are reservoirs for various pathogens, the contact between these two swine groups has important implications for disease transmission in the wildlife-livestock interface. This study provides a foundation to design targeted, cost-effective disease surveillance and risk mitigation programs in regions at highest risk for wild- domestic pig contact and can serve as a template for similar efforts nationwide.

Moreover, the results of this study provide a framework to create an outreach extension program and inform all stakeholders (e.g., farmers, government agencies) that may be called upon to respond to future zoonotic or TAD outbreaks, such as ASF. The results of this study, despite limitations, can provide important information to stakeholders and organizations that handle swine diseases or public health problems originating from any swine group in California.

Acknowledgements: The authors would like to thank the following people and organizations for their support and assistance: Robert Hijmans for Maximum Entropy expertise; Michele Tobias at UC Davis for QGIS assistance; Robert Johnson and Shane Feirer at UC ANR for building the online survey; Kristi Cripe and Craig Stowers at CDFW for hunting tag data and feral pigs information; Jim Thorne and Michele Stern for PRISM data; Jasmine Torres for online farm searches and big thanks to our feral pig experts: Dana Nelson, Rebecca Milhalco, Hector Webster, John Harper, and Roger Baldwin.

Author Contributions

Conceptualization: Laura Patterson, Alda Pires and Beatriz Martínez-López

Data curation: Laura Patterson

Funding acquisition: Alda Pires and Laura Patterson

Methodology: Laura Patterson, Jaber Belkhiria and Beatriz Martínez-López

Project administration: Alda Pires and Laura Patterson

Writing: Laura Patterson

Review & Editing: Laura Patterson, Alda Pires, Jaber Belkhiria and Beatriz Martínez-López

References:

1. Honeyman MS, Pirog RS, Huber GH, Lammers PJ, Hermann JR. The United States pork niche market phenomenon. J Anim Sci. 2014;84(8):2269–75.
2. Stephanie Strom. Demand grows for hogs that are raised humanely outdoors - NYTimes.com. New York Times [Internet]. 2014 Jan 20 [cited 2015 Apr 17]; Available from: http://www.nytimes.com/2014/01/21/business/demand-grows-for-hogs-that-are-raised-humanely.html?_r=0
3. McGlone JJ. Farm animal welfare in the context of other society issues: toward sustainable systems. Livest Prod Sci. 2001;72(1–2):75–81.
4. Scully T. Sustainable pork production without confinement becoming a reality - Farming [Internet]. Farming Magazine.com. 2017 [cited 2017 Sep 30]. Available from: https://www.farmingmagazine.com/livestock/pigs/sustainable-pork-production-without-confinement/
5. Davies PR. Intensive swine production and pork safety. Foodborne Pathog Dis. 2011;8(2):189–201.
6. Pedersen K, Quance CR, Robbe-Austerman S, Piaggio AJ, Bevins SN, Goldstein SM, et al. Identification of *Brucella suis* from feral swine in selected states in the USA. J Wildl Dis [Internet]. 2014;50(2):171–9. Available from: http://www.ncbi.nlm.nih.gov/pubmed/24484477
7. Pedersen K, Bevins SN, Schmit BS, Lutman MW, Milleson MP, Turnage CT, et al. Apparent prevalence of swine brucellosis in feral swine in the United States. Human-Wildlife Interact. 2012;6:38–47.
8. Hurley SP, Kliebenstein JB. A look at consumer willingness to pay for pork products with environmental attributes. Swine Research Report. 2000; 19.
9. Wheatley WP. Consumer preferences, premiums and the market for natural and organic pork: Locating a niche for small-scale producers. University of Minnesota Swine Center. 2001.
10. Corn JL, Cumbee JC, Barfoot R, Erickson GA. Pathogen exposure in feral swine populations geographically associated with high densities of transitional swine premises and commercial swine production. J Wildl Dis. 2009;45(3):713–21.
11. Martin BE, Sun H, Carrel M, Cunningham FL, Baroch JA, Hanson-Dorr KC, et al. US feral swine were exposed to both avian and swine influenza A viruses. Appl Environ Microbiol [Internet]. 2017;AEM.01346-17. Available from: http://aem.asm.org/lookup/doi/10.1128/AEM.01346-17
12. Wyckoff a C, Henke SE, Campbell T a, Hewitt DG, VerCauteren KC. Feral swine contact with domestic swine: a serologic survey and assessment of potential for disease transmission. J Wildl Dis. 2009;45(2):422–9.
13. Tabak MA, Piaggio AJ, Miller RS, Sweitzer RA, Ernest HB. Anthropogenic factors

predict movement of an invasive species. Ecosphere. 2017;8(6).
14. Schembri N, Hernandez-Jover M, Toribio JALML, Holyoake PK. On-farm characteristics and biosecurity protocols for small-scale swine producers in eastern Australia. Prev Vet Med [Internet]. 2015;118(1):104–16. Available from: http://dx.doi.org/10.1016/j.prevetmed.2014.11.008
15. Wu N, Abril C, Thomann A, Grosclaude E, Doherr MG, Boujon P, et al. Risk factors for contacts between wild boar and outdoor pigs in Switzerland and investigations on potential *Brucella suis* spill-over. BMC Vet Res [Internet]. 2012;8:116. Available from: http://www.pubmedcentral.nih.gov/articlerender.fcgi?artid=3464720&tool=pmcentrez&rendertype=abstract
16. Hartley M. Qualitative risk assessment of the role of the feral wild boar (*Sus scrofa*) in the likelihood of incursion and the impacts on effective disease control of selected exotic diseases in England. Eur J Wildl Res. 2010;56(3):401–10.
17. Tseng M, Fratamico PM, Bagi L, Manzinger D, Funk JA. Shiga toxin-producing *E. coli* (STEC) in swine: Prevalence over the finishing period and characteristics of the STEC isolates. Epidemiol Infect [Internet]. 2015;143(3):505–14. Available from: http://ovidsp.ovid.com/ovidweb.cgi?T=JS&CSC=Y&NEWS=N&PAGE=fulltext&D=emed12&AN=2014737130
18. Pearson HE, Toribio J-AL, Lapidge SJ, Hernández-Jover M. Evaluating the risk of pathogen transmission from wild animals to domestic pigs in Australia. Prev Vet Med [Internet]. 2016;123:39–51. Available from: http://www.sciencedirect.com/science/article/pii/S0167587715300878
19. Barton VCE, Black CC, Bolin CA, Breitmeyer RE, Bridges M, England SR, et al. Report of the Committee on Brucellosis. Proc Annu Meet U S Anim Health Assoc. 2005;68:132–5.
20. Berryman Institute. 2010 International Wild Pig Conference: Science & Management. In 2010. p. 1–44.
21. Pires AFA, Funk J A, Bolin C. Risk factors associated with persistence of *Salmonella* shedding in finishing pigs. Prev Vet Med [Internet]. 2014 Sep 1 [cited 2015 Jan 19];116(1–2):120–8. Available from: http://www.ncbi.nlm.nih.gov/pubmed/25005468
22. Navarro-Gonzalez N, Porrero MC, Mentaberre G, Serrano E, Mateos A, Cabal A, et al. *Escherichia coli* O157:H7 in wild boars (*Sus scrofa*) and Iberian ibex (*Capra pyrenaic*a) sharing pastures with free-ranging livestock in a natural environment in Spain. Vet Q. 2015;35(2):102–6.
23. Barth S, Geue L, Hinsching A, Jenckel M, Schlosser J, Eiden M, et al. Experimental evaluation of faecal *Escherichia coli* and Hepatitis E virus as biological indicators of contacts between domestic pigs and Eurasian wild boar. Transbound Emerg Dis. 2017;64(2):487–94.
24. Leiser OP, Corn JL, Schmit BS, Keim PS, Foster JT. Feral swine brucellosis in the United States and prospective genomic techniques for disease epidemiology. Vet Microbiol [Internet]. 2013;166(1–2):1–10. Available from: http://dx.doi.org/10.1016/j.vetmic.2013.02.025
25. Glazier N. Swine brucellosis outbreak in New York [Internet]. lancasterfarming.com. 2017 [cited 2017 Sep 30]. Available from: http://www.lancasterfarming.com/farming/dairy/swine-brucellosis-outbreak-in-new-york/article_d1b75a7e-a75f-5ab8-933a-b0df686aa82d.html

26. Hill DE, Dubey JP, Baroch J a., Swafford SR, Fournet VF, Hawkins-Cooper D, et al. Surveillance of feral swine for *Trichinella* spp. and *Toxoplasma gondii* in the USA and host-related factors associated with infection. Vet Parasitol [Internet]. 2014;205(3–4):653–65. Available from: http://linkinghub.elsevier.com/retrieve/pii/S0304401714004130
27. McKenna T. New York swine brucellosis. USDA. 2016.
28. Martínez-López B, Perez AM, Feliziani F, Rolesu S, Mur L, Sánchez-Vizcaíno JM. Evaluation of the risk factors contributing to the African swine fever occurrence in Sardinia, Italy. Front Microbiol. 2015;6(MAR):1–10.
29. Pineda-Krch M, O'Brien JM, Thunes C, Carpenter TE. Potential impact of introduction of foot-and-mouth disease from wild pigs into commercial livestock premises in California. Am J Vet Res. 2010;71(1):82–8.
30. Mur L, Martínez-López B, Costard S, de la Torre A, Jones B a, Martínez M, et al. Modular framework to assess the risk of African swine fever virus entry into the European Union. BMC Vet Res [Internet]. 2014;10:145. Available from: http://www.biomedcentral.com/1746-6148/10/145
31. Sur J-H. How far can African swine fever spread? J Vet Sci. 2019;20(4):1–5.
32. Joka FR, Van Gils H, Huang L, Wang X. High probability areas for ASF infection in china along the russian and korean borders. Transbound Emerg Dis [Internet]. 2018;(July 2018). Available from: http://doi.wiley.com/10.1111/tbed.13094
33. USDA APHIS. USDA Statement on confirmation of African swine fever in the Dominican Republic [Internet]. 2021 [cited 2021 Aug 7]. Available from: https://www.aphis.usda.gov/aphis/newsroom/news/sa_by_date/sa-2021/asf-confirm
34. Beasley JC, Ditchkoff SS, Mayer JJ, Smith MD, Vercauteren KC. Research priorities for managing invasive wild pigs in North America. J Wildl Manage. 2018;82(4):674–81.
35. Robbins J. Feral Pigs Roam the South. Now even Northern States aren't safe. Ecol Evol [Internet]. 2019 Dec 16 [cited 2019 Dec 19]; Available from: https://www.nytimes.com/2019/12/16/science/feral-pigs-canada-texas.html
36. USDA APHIS Veterinary Services. Spatial assessment of domestic and feral swine population data to identify information gaps and areas of potential contact between populations. 2015;(May):1–3. Available from: https://www.aphis.usda.gov/animal_health/nahms/swine/downloads/swine2012/FeralSwine1.pdf
37. Lewis JS, Farnsworth ML, Burdett CL, Theobald DM, Gray M, Miller RS. Biotic and abiotic factors predicting the global distribution and population density of an invasive large mammal. Sci Rep [Internet]. 2017;7(February):44152. Available from: http://www.nature.com/articles/srep44152
38. Snow NP, Jarzyna MA, Vercauteren KC. Interpreting and predicting the spread of invasive wild pigs. J Appl Ecol. 2017;
39. Oliver, W. & Leus K. *Sus scrofa*:The IUCN Red List of Threatened Species [Internet]. e.T41775A10559847. 2008 [cited 2017 Oct 30]. p. RLTS.T41775A10559847.en. Available from: http://www.iucnredlist.org/details/41775/0
40. Waithman JD. Guide to hunting wild pigs in California. 2001;(August):3–41.
41. Waithman JD, Sweitzer RA, VanVuren D, Drew JD, Brinkhaus AJ, Gardner IA. Range expansion, population sizes, and management of wild pigs in California. J Wildl Manage. 1999;63(1):298–308.
42. Corn J. National Feral Swine Mapping System. 1982.

43. USDA APHIS | National Feral Swine Program (NFSP) [Internet]. Available from: https://www.aphis.usda.gov/aphis/resources/pests-diseases/feral-swine/sa-fs-history
44. Oganesyan a. S, Petrova ON, Korennoy FI, Bardina NS, Gogin a. E, Dudnikov S a. African swine fever in the Russian Federation: Spatio-temporal analysis and epidemiological overview. Virus Res [Internet]. 2013;173(1):204–11. Available from: http://dx.doi.org/10.1016/j.virusres.2012.12.009
45. Wyckoff a. C, Henke SE, Campbell T a., Hewitt DG, Vercauteren KC. Movement and habitat use of feral swine near domestic swine facilities. Wildl Soc Bull. 2012;36(1):130–8.
46. McClure ML, Burdett CL, Farnsworth ML, Lutman MW, Theobald DM, Riggs PD, et al. Modeling and mapping the probability of cccurrence of invasive wild pigs across the contiguous United States. PLoS One. 2015;10(8):e0133771.
47. Barth SA, Blome S, Cornelis D, Pietschmann J, Laval M, Maestrini O, et al. Faecal *Escherichia coli* as biological indicator of spatial interaction between domestic pigs and wild boar (*Sus scrofa*) in Corsica. Transbound Emerg Dis. 2018;(June 2017).
48. Rutten A, Casaer J, Swinnen KRR, Herremans M, Leirs H. Future distribution of wild boar in a highly anthropogenic landscape: Models combining hunting bag and citizen science data. Ecol Modell [Internet]. 2019 Nov 1 [cited 2019 Sep 17];411:108804. Available from: https://www.sciencedirect.com/science/article/pii/S0304380019303126
49. Abdrakhmanov SK, Mukhanbetkaliyev YY, Korennoy FI, Sultanov AA, Kadyrov AS, Kushubaev DB, et al. Maximum entropy modeling risk of anthrax in the Republic of Kazakhstan. Prev Vet Med. 2017;144:149–57.
50. Elith J, Phillips SJ, Hastie T, Dudík M, Chee YE, Yates CJ. A statistical explanation of MaxEnt for ecologists. Divers Distrib [Internet]. 2011 Jan [cited 2019 Nov 21];17(1):43–57. Available from: http://doi.wiley.com/10.1111/j.1472-4642.2010.00725.x
51. Belkhiria J, Alkhamis MA, Martínez-López B. Application of species distribution modeling for avian influenza surveillance in the United States considering the North America migratory flyways. Sci Rep [Internet]. 2016;6(August):33161. Available from: http://www.nature.com/articles/srep33161
52. Baldwin RA. Use of maximum entropy modeling in wildlife research. Entropy. 2009;11(4):854–66.
53. LaHue NP, Baños JV, Acevedo P, Gortázar C, Martínez-López B. Spatially explicit modeling of animal tuberculosis at the wildlife-livestock interface in Ciudad Real province, Spain. Prev Vet Med [Internet]. 2016;128:101–11. Available from: http://linkinghub.elsevier.com/retrieve/pii/S016758771630126X
54. Phillips SJ, Anderson RP, Schapire RE. Maximum entropy modeling of species geographic distributions. Ecol Modell. 2006;190(3–4):231–59.
55. Phillips S. A Brief Tutorial on Maxent. AT&T Res. 2008;3:1–38.
56. Franklin J. Mapping Species Distributions. 2010. 1–320 p.
57. Hijmans RJ, Elith J. Species distribution modeling with R. R CRAN Proj [Internet]. 2015;79 pp. Available from: http://www.idg.pl/mirrors/CRAN/web/packages/dismo/vignettes/sdm.pdf%5Cnpapers://e21e3140-ccc7-4142-911c-e74fc5ceecf7/Paper/p10162
58. Merow C, Smith MJ, Silander J a. A practical guide to MaxEnt for modeling species' distributions: what it does, and why inputs and settings matter. Ecography (Cop) [Internet]. 2013;36(10):1058–69. Available from: http://doi.wiley.com/10.1111/j.1600-

0587.2013.07872.x

59. Hijmans RJ. Species Distribution Modeling. p. https://rspatial.org/raster/sdm/5_sdm_models.html.

60. Cavalcante T, de Souza Jesus A, Rabelo RM, Messias MR, Valsecchi J, Ferraz D, et al. Niche overlap between two sympatric frugivorous Neotropical primates: improving ecological niche models using closely-related taxa. Biodivers Conserv. 2020;29(8):2749–63.

61. Alexander NS, Massei G, Wint W. The European distribution of *Sus scrofa*. Model outputs from the project described within the poster – Where are all the boars? An attempt to gain a continental perspective. Open Heal Data. 2016;4:1–6.

62. Chen X, Lei Y. Effects of sample size on zccuracy and stability of species distribution models: a comparison of GARP and MaxEnt. Lect Notes Electr Eng. 2012;125 LNEE(VOL. 2).

63. Stockwell DRB, Peterson AT. Effects of sample size on accuracy of species distribution models. Ecol Modell. 2002;148(1):1–13.

64. Hernandez PA, Graham CH, Master LL, Albert DL. The effect of sample size and species characteristics on performance of different species distribution modeling methods. Ecography (Cop). 2006;29(5):773–85.

65. Garza SJ, Tabak MA, Miller RS, Farnsworth ML, Burdett CL. Abiotic and biotic influences on home-range size of wild pigs (*Sus scrofa*). J Mammal. 2018;99(1):97–107.

66. Holmstrom L. Feral swine and foreign and emerging animal diseases. In: National Center for Foreign Animal & Zoonotic Disease Defense. 2013.

67. Conservancy TN. An sssessment of the known and potential impacts of feral pigs (*Sus scrofa*) in and near San Diego County with management recommendations. Conserv Biol Inst. 2009;(October).

68. Sweitzer R, Vuren D Van. Estimating sizes of wild pig populations in the north and central coast regions of California. J Wildl … [Internet]. 2000;64(2):531–43. Available from: http://www.jstor.org/stable/3803251

69. Pittiglio C, Khomenko S, Beltran-Alcrudo D. Wild boar mapping using population-density statistics: From polygons to high resolution raster maps. PLoS One [Internet]. 2018;13(5):1–19. Available from: http://dx.doi.org/10.1371/journal.pone.0193295

70. NASA. MODIS [Internet]. Available from: https://modis.gsfc.nasa.gov/data/

71. Broxton PD, Zeng X, Scheftic W, Troch PA. A MODIS-based global 1-km maximum green vegetation fraction dataset. J Appl Meteorol Climatol. 2014;53(8):1996–2004.

72. Didan K, Munoz AB, Solano R, Huete A. MODIS Vegetation Index User's Guide (MOD13 Series) Version 3.0 Ccollection 6). 2015;2015(May):38.

73. WorldClim [Internet]. Available from: https://www.worldclim.org

74. O'Donnell MS, Ignizio DA. Bioclimatic Predictors for supporting ecological applications in the conterminous United States. US Geol Surv Data Ser 691. 2012;10.

75. QGIS Development Team. QGIS Geographic Information System [Internet]. QGIS Geographic Information System. Open Source Geospatial Foundation Project; 2016. Available from: https://qgis.org/en/site/

76. RStudio: Integrated Development for R. RStudio [Internet]. Boston, MA: RStudio, PBC; 2019. Available from: http://www.rstudio.com/

77. Hijmans RJ, Phillips S, And JL, Elith A. dismo: Species Distribution Modeling. R package version 1.3-3. 2019.

78. Hijmans RJ. raster: Geographic data analysis and modeling. R package version 3.4-10. 2019.
79. Pebesma EJ, R.S. Bivand. sp: Classes and methods for spatial data in R [Internet]. R News 5 (2),; 2005. Available from: https://cran.r-project.org/doc/Rnews/
80. Hijmans RJ. Introduction to the 'raster' package (version 1.9-63). 2012;(2008):1–26.
81. Morales NS, Fernández IC, Baca-González V. MaxEnt's parameter configuration and small samples: Are we paying attention to recommendations? A systematic review. PeerJ. 2017;2017(3):1–16.
82. Gallardo B, Zieritz A, Aldridge DC. The importance of the human footprint in shaping the global distribution of terrestrial, freshwater and marine invaders. PLoS One. 2015;10(5):1–17.
83. Jack Mayer. Feral hog behavior [Internet]. extension.org. 2012. Available from: https://articles.extension.org:443/pages/64381/feral-hog-behavior
84. Slater H, Michael E. Predicting the current and future potential distributions of lymphatic filariasis in africa using maximum entropy ecological niche modelling. PLoS One. 2012;7(2).
85. Hijmans RJ. Cross-validation of species distribution models : removing spatial sorting bias and calibration with a null model. Ecol Soc Am Stable [Internet]. 2012;93(3):679–88. Available from: ww.jstor.org/stable/23143954
86. Google LLC. Google Earth Pro v. 7.3.2 [Internet]. Mountain View, CA; 2018. Available from: http://www.earth.google.com
87. ESRI. Survey 123 v3.6 [Internet]. Redlands, CA; 2018. Available from: www.esri.com
88. Kay SL, Fischer JW, Monaghan AJ, Beasley JC, Boughton R, Campbell TA, et al. Quantifying drivers of wild pig movement across multiple spatial and temporal scales. Mov Ecol [Internet]. 2017;5(1):14. Available from: http://movementecologyjournal.biomedcentral.com/articles/10.1186/s40462-017-0105-1
89. Holmstrom L. Identifying and optimizing prevention and control strategies against the spread of viral pathogens within U.S. feral swine populations. 2013;
90. Corn JL, Jordan TR. Development of the national feral swine map, 1982–2016. Wildl Soc Bull. 2017;41(4):758–63.
91. Bosch J, Mardones F, Pérez A, De la Torre A, Muñoz MJ. A maximum entropy model for predicting wild boar distribution in Spain. Spanish J Agric Res [Internet]. 2014;12(4):984. Available from: http://revistas.inia.es/index.php/sjar/article/view/5717
92. Lobo JM, Jiménez-valverde A, Real R. AUC: A misleading measure of the performance of predictive distribution models. Glob Ecol Biogeogr. 2008;17(2):145–51.
93. USDA-APHIS-VS-CEAH-NAHMS. Swine 2012: Reference of Management Practices on small-enterprise swine operations in the United States. 2014.
94. Miller RS, Sweeney SJ, Slootmaker C, Grear DA, Di Salvo PA, Kiser D, et al. Cross-species transmission potential between wild pigs, livestock, poultry, wildlife, and humans: implications for disease risk management in North America. Sci Rep [Internet]. 2017;7(1):1–14. Available from: http://dx.doi.org/10.1038/s41598-017-07336-z
95. Swain DL, Langenbrunner B, Neelin JD, Hall A. Increasing precipitation volatility in twenty-first-century California. Nat Clim Chang [Internet]. 2018;8(5):427–33. Available from: http://dx.doi.org/10.1038/s41558-018-0140-y
96. Watts N, Adger WN, Agnolucci P, Blackstock J, Byass P, Cai W, et al. Health and climate change: policy responses to protect public health. Lancet [Internet]. 2015;6736(15).

Available from: http://linkinghub.elsevier.com/retrieve/pii/S0140673615608546
97. Pitesky M, Acosta S. Poultry Ponderings. UCDavis Veterinary Medicine Poultry Ponderings. 2020;Edition 18:1–5.

Supporting Information

S1 Table: The analysis of variable contribution table provided estimates of the relative contribution of each variable to the final MaxEnt model.

S1 Fig: Jackknife results for final MaxEnt model. The Jackknife graph indicated importance of key variables: BIO6 was the minimum temperature of the coldest month, AVGMODIS was the annual maximum green vegetation fraction, BIO13 was the precipitation of the wettest month, BIO15 was the variation of annual precipitation and elevation.

S2 Fig: MaxEnt response curves for the five significant variables used in final MaxEnt model. The response curves generated with MaxEnt show the predicted probability of suitable feral pig habitat for each individual variable, for each level of the predictor. The minimum temperature of the coldest month (BIO6), the annual maximum green vegetation fraction (AVGMODIS), the precipitation of the wettest month (BIO13), the variation of annual precipitation (BIO15) and elevation.

Figure 1

Click here to access/download;Figure;Fig1.tif

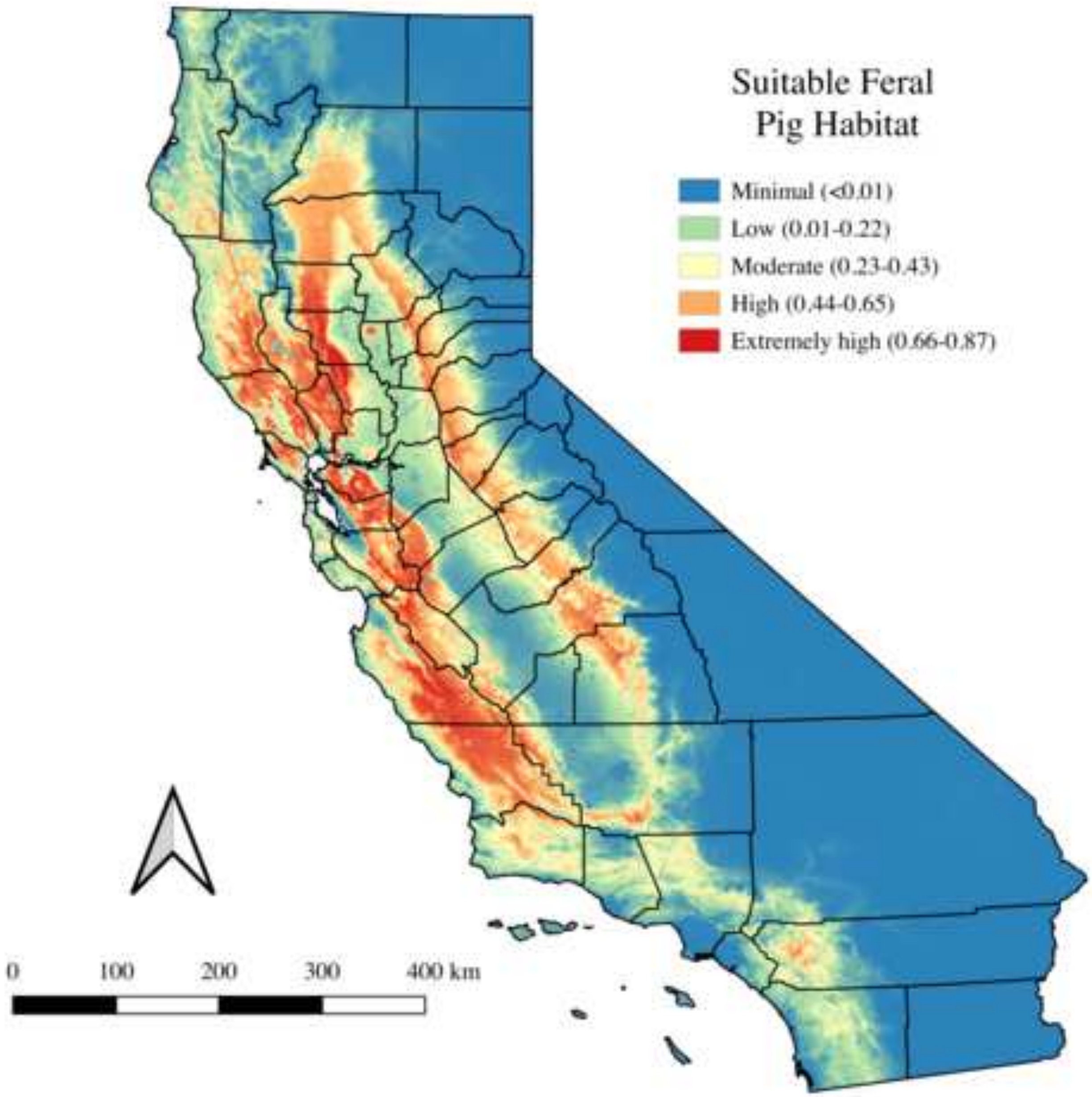

Figure 2

Click here to access/download;Figure;Fig2.tif

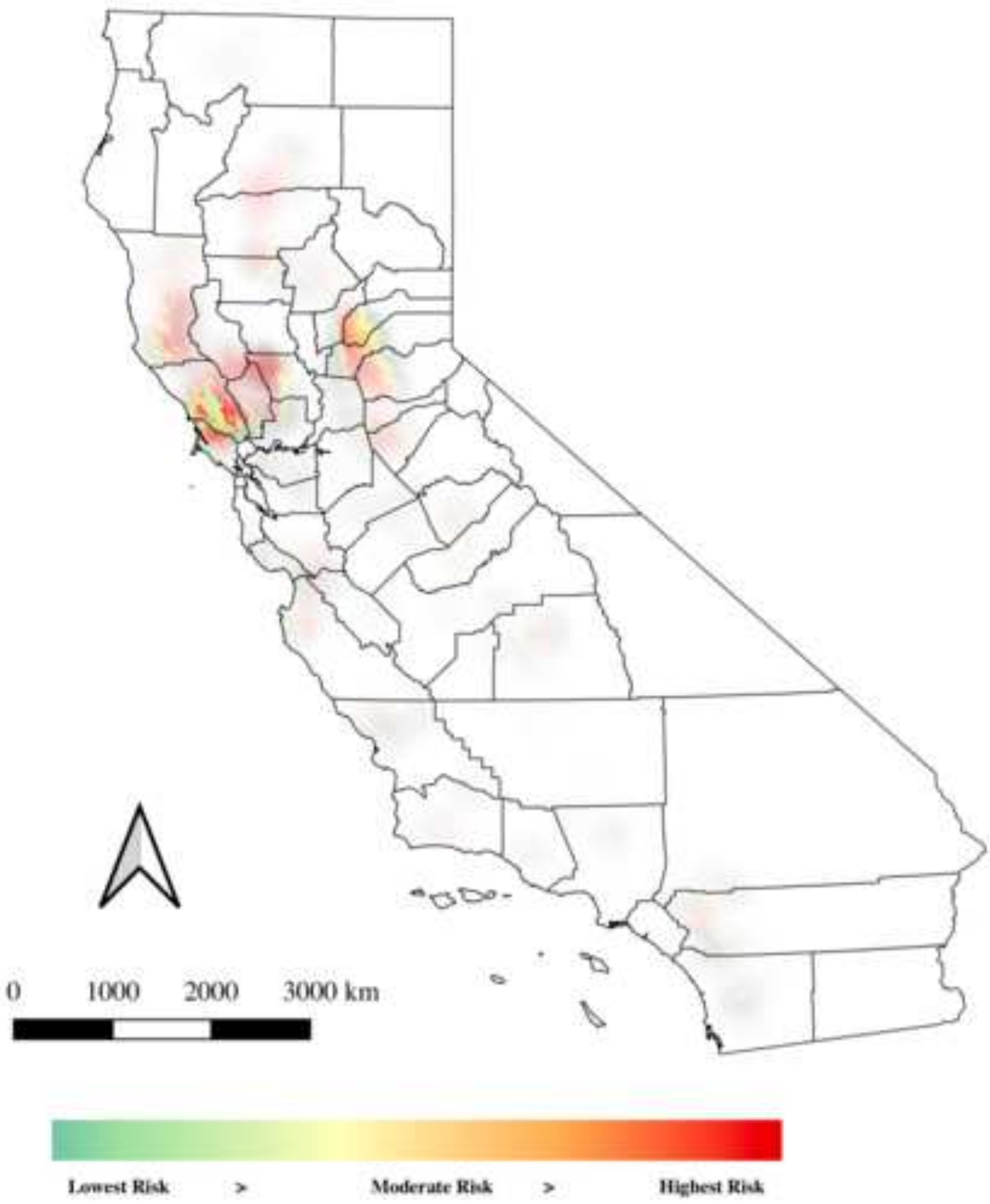

Supplement: Attachment — Submitted filename: PONE-D-21-36324 (1)_VB.pdf [file pone.0270500.s005.pdf]
