# Supplementary material for: Identification of high-risk contact areas between feral pigs and outdoor-raised pig operations in California: Implications for disease transmission in the wildlife-livestock interface
Source: PLoS One. 2022 Jun 28;17(6):e0270500. doi: 10.1371/journal.pone.0270500 (PMC9239460; doi:10.1371/journal.pone.0270500)
Supplement: Attachment — Submitted filename: Response to Editor R2.docx [file pone.0270500.s006.docx]

**PONE-D-21-36324R1**
Identification of high-risk contact areas between feral pigs and outdoor-raised pig operations in California: implications for disease transmission in the wildlife-livestock interface

Dear Dr. Pires,

We've checked your submission and before we can proceed, we need you to address the following issues:

1. Please upload a Response to Reviewers letter which should include a point by point response to each of the points made by the Editor and / or Reviewers. (This should be uploaded as a 'Response to Reviewers' file type.) Please follow this link for more information: <http://blogs.PLOS.org/everyone/2011/05/10/how-to-submit-your-revised-manuscript/>
**AR1**: We apologize for this oversight. ‘ Response to Reviewers’ was uploaded.

2. Thank you for including your ethics statement on the online submission form:  "This study used secondary data, doesn't included field work, nor animal primary data. An IRB protocol was reviewed and determined to be exempted by the UC Davis IRB. See Manuscript lines 180-183: The survey instrument and protocols were reviewed and exempted by the Institutional Review Board (IRB) of the University of California-Davis (No. 1180798-1)."
To help ensure that the wording of your manuscript is suitable for publication, would you please also add this statement at the beginning of the Methods section of your manuscript file.

**AR2**: The ethics statement was modified and moved to the beginning of Materials & Methods section.

3. Please note that PLOS ONE is unable to publish previously copyrighted maps or satellite images, or images created using proprietary data. For these reasons, we cannot publish images generated by software which copyrights their output (such as Google Maps, Street View, and Earth). In order to use these images in your submission, we require explicit permission from the copyright owner to publish the figures under the CC BY 4.0 license.
At this time, please kindly clarify the following regarding Figures 1, 2, and 4:
a) Where did the authors obtain the maps, satellite images, basemaps, shapefiles, map data, etc. in Figures 1, 2, and 4?  **AR3:** Please see below a detailed explanation of the origin/development of the figures.

**Figure 1:** CDFW Hunting tag maps are publically available online, not copyrighted. CDFW, kindly provided us with the data needed for this study.

**Figure 2:** This is an original product developed by the authors. The MaxEnt map was created by the first author by using publically available layers online. The links to these layers are listed in Table 1 and can easily be downloaded by others. Additionally, the reference section also contains citations and links to each layer used in MaxEnt. See a similar MaxEnt project published by the second author in PlosOne.(1) In this PlosOne publication, copyrights were not requested, only citations were required to be listed. Once the layers were downloaded, the first author standardized them to the same extent (i.e., shape of California), resolution (i.e., 270m x 270m) and projection (i.e., California Albers (meters)). All standardized layers were converted to rasters before using in model building steps in R. Additionally, we included the final rasters for the five significant variables in the final model.

1.	Belkhiria J, Hijmans RJ, Boyce W, Crossley BM, Martínez-López B. Identification of high risk areas for avian influenza outbreaks in California using disease distribution models. PLoS One. 2018;13(1):1–15.

**Figure 4:** This is an original product developed by the authors. This was done overlapping the MaxEnt model, built by the first author (see layer information above for Fig 1 and 2) and outdoor-raised pig operations that were collected by the first author and last author’s lab staff.

b) Please state whether the map/satellite images have been previously copyrighted to your knowledge.

**AR4:** Climatic data was obtained from worldclim.org and this was cited in the manuscript. Worldclim layers are publically available, funede with non profit and federal grants and not copyrighted but available to all in science who wish to use them for research projects.

“AVGMODIS”data, was downloaded from a NASA site. There is a data user guide the NASA site wrote with Boston University here: <https://www.bu.edu/lcsc/data-documentation/>

Briefly it says: “source code and data products are available free of charge to the public. Source code can be accessed via our [GitHub account](https://github.com/BU-LCSC). MODIS data products are archived and available via FTP from the [Land Processes Distributed Active Archive Center](https://lpdaac.usgs.gov/) (DAAC) at EROS Data Center.  Other data sets created through funding from NASA’s ABoVE project and the NSF’s Macrosystems Biology Funding are available from the Oak Ridge National DAAC.” Also on NASA’s earth data site they say “NASA promotes the full and open sharing of all data with the research and applications communities, private industry, academia, and the general public.”

c) If any of the [map/satellite] images have been previously copyrighted, we require specific consent from the copyright holder to publish these images in PLOS ONE, under the CC BY 4.0 license. To seek permission from the copyright owner to publish your map figures under the specific Creative Commons Attribution License (CCAL), CC BY 4.0, please contact them with the following text and PLOS ONE Request for Permission form (<http://journals.plos.org/plosone/s/file?id=7c09/content-permission-form.pdf>):

**AR5:** All data used fro this study are publically available online to be used by scientists and students worldwide.

“I request permission for the open-access journal PLOS ONE to publish XXX under the Creative Commons Attribution License (CCAL) CC BY 4.0 (<http://creativecommons.org/licenses/by/4.0/>). Please be aware that this license allows unrestricted use and distribution, even commercially, by third parties. Please reply and provide explicit written permission to publish XXX under a CC BY license.”

Please upload the granted permission to the manuscript as a Supporting Information file. In the figure caption of the copyrighted figure, please include the following text: “Republished from [ref] under a CC BY license, with permission from [name of publisher], original copyright [original copyright year].”

Please note that RightsLink permission forms often impose use restrictions that are incompatible with our CC BY 4.0 license, and we are therefore unable to accept these permissions. For this reason, we strongly recommend contacting copyright holders with the PLOS ONE Request for Permission form.

If you are unable to obtain permission from the copyright holder, please either A) remove the figure or B) supply a replacement figure that complies with the CC BY 4.0 license. Please check copyright information on all replacement figures and update the figure caption with source information.

The following resources for replacing copyrighted map figures may be helpful:

USGS National Map Viewer (<http://viewer.nationalmap.gov/viewer/>)
USGS Earth Resources Observatory and Science (EROS) Center ([http://eros.usgs.gov/#](http://eros.usgs.gov/))
The Gateway to Astronaut Photography of Earth (<https://eol.jsc.nasa.gov/>)
Maps at the CIA (<https://www.cia.gov/library/publications/the-world-factbook/docs/refmaps.html>)
NASA Earth Observatory (<http://earthobservatory.nasa.gov/>)
Landsat (<http://landsat.visibleearth.nasa.gov/>)
Natural Earth (<http://www.naturalearthdata.com/>)

4. We note that your Data Availability Statement reads: "OPO data cannot be shared publicly, as they contain GPS coordinates and participants provided locations based on confidentiality. Feral pig hunting tags are provided in the text as Fig1. Predictor layers used to build a MaxEnt model are publicly available online, but rasters included in the final MaxEnt model are included in KML format as supplementary files." Please respond to the following prompts:

A. We note that you have stated that OPO data cannot be shared publicly. At this time please provide: (1) The name of the institutional body who has imposed the restrictions upon these data (e.g., a Research Ethics Committee or Institutional Review Board, etc.) and (2) direct, non-author contact information (preferably email) for the body imposing the restrictions upon the data or another non-author contact to which data access requests can be sent.

**AR6:** The project protocol, including survey and cover -letter, was reviewed and determined to be exempted by the Institutional Review Board (IRB) of the University of California-Davis (No. 1180798-1’. The farm location and farm ID is confidential. There was a written condifentiality agreement (in the cover-letter of this survey) stating that all provided information is kept confidential, and all data would be reported as aggregated information.

B. Regarding the predictor layers to build your model, please provide a link or DOI to the predictor layers used. Please also specify if these are third party data. If so please explain how others can access these datasets and confirm that others would be able to access these data in the same manner as the authors. Please also confirm that the authors did not have any special access privileges that others would not have. We will update your Data Availability statement upon your response.

**AR6:** The links are provided in Table 1. Citations are listed in the References. DOI numbers are not available. Previous PlosOne papers have also just listed the links to publically available layers downloaded from online sites Additionally, we included the final rasters for the five significant variables in the final modelm if someone wants to build the same MaxEnt model.

We've returned your manuscript to your account. Please resolve these issues and resubmit your manuscript within 21 days. If you need more time, please email the journal office at [plosone@plos.org](mailto:plosone@plos.org). We are happy to grant extensions of up to one month past this due date. If we do not hear from you within 21 days, we will withdraw your manuscript.

Please log on to PLOS Editorial Manager at <https://www.editorialmanager.com/pone/> to access your manuscript. You will find your manuscript in the 'Submissions Sent Back to Author' link under the New Submissions menu. Be sure to remove your previous manuscript file if you are uploading a new file in response to these requests. After you've made the changes requested above, please be sure to view and approve the revised PDF after rebuilding the PDF to complete the resubmission process.

We are requesting these changes to comply with the PLOS ONE submission guidelines (<https://journals.plos.org/plosone/s/submission-guidelines>). Please note that we won't send your manuscript for review until you have resolved the above requests. 

Thank you for submitting your work to PLOS ONE and supporting our mission of Open Science.


Kind regards,

Rose Ann Joyce Sagun Puetes
PLOS ONE
